# Role of NADPH Oxidases in Blood–Brain Barrier Disruption and Ischemic Stroke

**DOI:** 10.3390/antiox11101966

**Published:** 2022-09-30

**Authors:** Marina S. Hernandes, Qian Xu, Kathy K. Griendling

**Affiliations:** Division of Cardiology, Department of Medicine, Emory University, Atlanta, GA 30322, USA

**Keywords:** NADPH oxidase, blood–brain barrier, ischemic stroke

## Abstract

NADPH oxidases (Nox) are one of the main sources of reactive oxygen species (ROS) in the central nervous system (CNS). While these enzymes have been shown to be involved in physiological regulation of cerebral vascular tone, excessive ROS produced by Nox1-5 play a critical role in blood–brain barrier (BBB) dysfunction in numerous neuropathologies. Nox-derived ROS have been implicated in mediating matrix metalloprotease (MMP) activation, downregulation of junctional complexes between adjacent brain endothelial cells and brain endothelial cell apoptosis, leading to brain microvascular endothelial barrier dysfunction and consequently, increases in BBB permeability. In this review, we will highlight recent findings on the role played by these enzymes in BBB disruption induced by ischemic stroke.

## 1. Introduction: NADPH Oxidases in the Brain

The NADPH oxidase (Nox) enzyme family of superoxide (O_2_^•−^)- and hydrogen peroxide (H_2_O_2_)-producing proteins is a major source of reactive oxygen species (ROS) in the central nervous system (CNS). The Nox family is comprised of seven homologs: Nox1, Nox2 (gp91^phox^), Nox3, Nox4, Nox5, DUOX1, and DUOX2, five of which have been found in the CNS (Nox1, Nox2, Nox3, Nox4, and Nox5) [1,2,3]. Several oxidases have regulatory subunits that are essential for their activity. The main biological function of NADPH oxidases is the production of superoxide via a single electron reduction. Because of its negative charge, superoxide is not able to diffuse across biological membranes and its effect is mainly local. Superoxide can spontaneously dismutate to hydrogen peroxide (H_2_O_2_), but this reaction can be enzymatically catalyzed by superoxide dismutases (SOD). H_2_O_2_, in turn, is more stable when compared to superoxide, and is able to cross biological membranes [4]. Nox4 is somewhat unique among the NADPH oxidases, since the major detectable product is H_2_O_2_, even though it initially produces superoxide [5].

The physiological functions of Nox family members are extremely diverse and include cell growth, differentiation, apoptosis, cytoskeletal remodeling and senescence [6,7]. These enzymes also play an essential role in host defense: superoxide produced by Nox2 is required for the respiratory burst that occurs in phagocytes [8]. In addition, more specialized functions of Noxes include neuronal development and axonal outgrowth controlled by Nox2 [9], the iodination of thyroid hormone mediated by Duox2 [10], and oligodendrocyte differentiation and otoconium formation in the inner ear mediated by Nox3 [11,12].

Overactivation of specific Nox isoforms has been linked to several CNS pathologies including Alzheimer’s disease (AD), Parkinson’s disease (PD), amyotrophic lateral sclerosis (ALS), stroke, multiple sclerosis (MS), and traumatic brain injury (TBI). In this review, we will briefly present and describe the NADPH oxidases relevant to CNS pathophysiology and explore our current understanding of the role played by these enzymes in blood–brain barrier (BBB) disruption induced by ischemic stroke.

## 2. Nox Proteins in CNS Signal Transduction

### 2.1. Nox1

**Figure 1:**
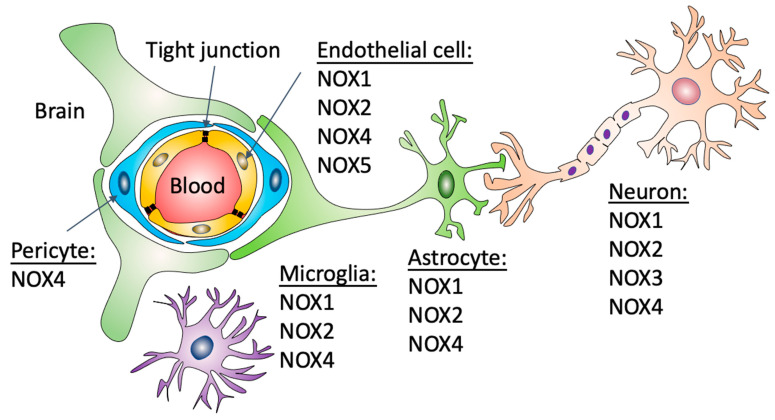
Expression of NADPH oxidases in different cell types that form the BBB. The BBB is formed by capillary brain endothelial cells surrounded by basal lamina, pericytes and astrocytic perivascular endfeet. Brain capillaries are also surrounded by microglial cells and neuronal processes. Nox1 and 2 have been described in endothelial cells, astrocytes, neurons and microglial cells. Nox4 has been described in endothelial cells, astrocytes, neurons, microglial cells and pericytes. Nox3, in contrast, has only been described in neurons and Nox5 in brain endothelial cells.

Nox1 associates with the membrane subunit p22phox and it is fully activated by forming a complex with its cytosolic activators p47phox and p67phox, or its homologs NoxO1 and NoxA1, respectively, and GTP-Rac [13]. In the CNS, Nox1 is expressed in neurons [1], astrocytes [14], microglia [15], and brain endothelial cells [16] (Figure 1). ROS produced by activation of Nox1 have been implicated in neuronal cell death. AAV-mediated Nox1 downregulation increased neuronal survival in peri-infarct regions and enhanced functional recovery after stroke induction in rats [17]. In a 6-hydroxydopamine (6-OHDA)-induced PD model, treatment with fucoidan, which interferes with Nox1 signaling, attenuated neuronal loss in the substantia nigra pars compacta [18]. Mechanistically, studies showed that Nox1-induced neuronal cell death is mediated by protein kinase C δ (PKC δ) [19]. In Zn^2+^-mediated neurodegeneration, protein kinase C (PKC)-induced Nox1 activation mediates transient receptor potential melastatin 2 (TRPM2)-dependent intercellular Ca^2+^ overload via adenosine diphosphate ribose (ADPR) production, resulting in apoptosis [20]. A recent study also demonstrated that glutamate-induced ROS production, lipid peroxidation and hippocampal cell death is mediated by glucose-dependent insulinotropic polypeptide (GIP) and mitogen-activated protein kinase (MAPK)-induced Nox1 activation [21].

A link between Nox1 activation and neuroinflammation has also been demonstrated. In an in vivo model of traumatic brain injury (TBI), Nox1-induced transforming growth factor-β1 (TGF-β1) signaling phosphorylated Smad2 and Smad3, which then mediated secretion of inflammatory cytokines, including interleukin-1β (IL-1β) and tumor necrosis factor alpha (TNF-α) [22]. In microglial cells, Nox1-derived ROS enhance expression of inducible nitric oxide synthase and secretion of IL-1β, which has been shown to mediate loss of synaptic proteins in vivo [15].

### 2.2. Nox2

Similar to Nox1, Nox2 activation depends on the assembly of several cytosolic regulatory subunits. The Nox2 complex is formed of the membrane subunits Nox2 (gp91phox) and p22phox and is activated by the phosphorylation and translocation of the cytosolic subunit p47phox, followed by p67phox binding to p47phox. Rac1 is also required for activation [13,23].

Nox2 has been found in neurons, astrocytes, microglia [24,25], oligodendrocytes [26] and brain endothelial cells [27] (Figure 1). A substantial correlation between Nox2 activation and CNS pathologies has been extensively demonstrated. Neuronal Nox2 levels are elevated after spared nerve injury and the resulting oxidative stress promotes hyperexcitability of dorsal root ganglion neurons and mechanical allodynia [28]. In TBI patients, Nox2 was found upregulated in cortical parvalbumin-positive interneurons, which was also observed in a murine TBI model. The consequent oxidative damage led to loss of GABAergic-parvalbumin neurons and glutamatergic excitoxicity [29]. In cultured cortical neurons, N-methyl-D-aspartate (NMDA) receptor-induced activation of Nox2 triggers oxidative stress in neighboring neurons and astrocytes by a mechanism that involves extracellular release of superoxide [30]. Neuronal Nox2 signal transduction pathways not only include NMDA receptor activation but also Src/phosphoinositide 3-kinase (PI3K)/Akt (protein kinase B) and caspase 3-dependent neuronal apoptosis [20].

In microglial cells, increased ROS production by Nox2 plays a role in aging-associated Aβ deposition, protein tyrosine nitration and IL-1β generation [25]. Microglia proliferation appears to be Nox dependent, as the non-specific Nox inhibitors DPI and apocynin blocked IL-1β and TNF-α-stimulated proliferation [31], although these inhibitors do not directly implicate Nox2. These data are consistent with the concept that microglial Nox2 is essential to the CNS inflammatory response. Dopaminergic neurodegeneration induced by 6-OHDA, LPS and α-synuclein has been found to be regulated by microglial Nox2 activation [32,33]. More recently, activation of complement receptor 3 (CR3, aka macrophage-1 antigen (MAC1)), a microglia-specific pattern recognition receptor, has been identified as a regulator of Nox2 activation and dopaminergic neurodegeneration through a Src/Extracellular-signal regulated kinase (ERK)-dependent pathway [34]. Additional studies have also indicated an association between Nox2 activation and NF-ĸB and Rho-kinase activation in microglial motility, phagocytosis and inflammatory response [35].

### 2.3. Nox3

Nox3 mechanisms of activation are still controversial. Nox3 has been shown to be activated by the regulatory subunits p47phox and p67phox. In the same study, Nox3 was activated by NOXO1 in the absence of NOXA1 or p67phox [36]. Additional experimental evidence demonstrates that Nox3 forms a functional complex with p22phox and that p47phox and NOXO1 can enhance ROS production in the absence of any additional regulatory subunits [37].

Limited data are available on the role of Nox3 in the CNS. Nox3 is found in oligodendrocytes [11] and its expression is induced in neurons (Figure 1) after mechanical injury [24]. Earlier studies showed that Nox3 is expressed in the inner ear and that mutations in Nox3 lead to a head tilt phenotype. While hearing ability was not affected, histological evaluation of inner ear morphology revealed a complete lack of otoconia. Functional tests revealed abnormal performance in several motor coordination tests and lack of response to linear acceleration of the head with vestibular-evoked potentials, indicating a severe balance disorder resulting from mutations in Nox3 [12].

In oligodendrocytes, Nox3 and Nox5-derived ROS have been shown to contribute to oligodendrocyte differentiation through a mechanism that involves ERK1/2- and cyclic adenosine monophosphate (cAMP)-responsive element-binding protein (CREB), which may indicate that Nox3 plays an important role in CNS demyelinating pathologies [11]. More recently, it has been shown that the N64Y point mutation in Nox3 increases ROS production by mouse cerebellar granule cell precursors, leading to increases in proliferation through stimulation of the Sonic hedgehog (SHH) signaling pathway. Nox3-mediated increased granule cell proliferation impairs Purkinje cell development, leading to ataxia [38].

### 2.4. Nox4

In terms of its molecular organization, p22phox is essential to Nox4 activation, but traditional cytosolic factors are dispensable. Additional evidence demonstrated that polymerase-d interacting protein-2 (Poldip2) enhances its activity [39], but is not obligatory.

Nox4 is highly expressed in neurons [40], and has been found to be present in astrocytes [41], microglia [42], pericytes [43], and brain endothelial cells [40] (Figure 1). In neurons, Nox4 is involved in NMDA and α-amino-3-hydroxy-5-methyl-4-isoxazole propionic acid (AMPA) -induced H_2_O_2_ production and cytotoxicity [44]. In a humanized mouse model of tauopathy induced by brain delivery of AVV-Tau^P301L^, Nox4 depletion in neurons prevented cognitive decline and reduced neurotoxicity through modulation of the autophagy-lysosomal pathway and improved macroautophagy flux [45]. A role of Nox4 in autophagy has been proposed in additional studies [46]. Treatment with galantamine, which downregulates Nox4, prevents amyloid beta (Aβ)-mediated ROS accumulation and autophagy in cultured PC12 neurons [47].

A role for Nox4 in mediating neuroinflammation has also been described. Microglial cells stimulated with advanced oxidation protein products, biomarkers of oxidative stress, exhibited increased Nox4 expression. Nox4-derived ROS induced p38 mitogen-activated protein kinase (p38 MAPK) and c-jun N-terminal kinase (JNK) phosphorylation, subsequently triggering nuclear translocation of NF-κB p65 to induce pro-inflammatory cytokines [48]. The involvement of Nox4 in mediating the microglial janus kinase 2 (JAK2) and signal transducer and activator of transcription 3 (STAT3)-pathway and NLRP3 inflammasome activation in vivo and in vitro has also been described recently [49]. In addition, Parp3, a member of the Poly(ADP-ribose) polymerase (Parp) family, mediates Nox4-induced ROS production and impaired mammalian target of rapamycin complex 2 (mTorc2) activation leading to inefficient differentiation of post-natal neural stem/progenitor cells to astrocytes [50].

### 2.5. Nox5

Nox5 differs from other NADPH oxidases because it requires no additional regulatory subunits or p22phox but contains an N-terminal regulatory domain with four binding sites for Ca^2+^ (EF-hands). Upon Ca^2+^ binding, the regulatory domain exposes hydrophobic residues that bind to the C-terminal catalytic domain to activate Nox5 [51]. Calcium sensitivity is increased by calmodulin, which has been shown to significantly enhance ROS production [7].

Similar to Nox3, limited information is available on the role of Nox5 in the CNS. In humans, Nox5 has been shown to be broadly expressed in microvascular endothelial cells [52] (Figure 1). Because Nox5 is not expressed in rodents, a new mouse line expressing the human Nox5 gene has been recently created. Using this system, the authors showed in acute Nox5-dependent ROS production in organotypic hippocampal cultures stimulated with oxygen and glucose deprivation [53]. In a different study, using a knock-in system in which Nox5 is only expressed in endothelial cells, a positive correlation between Nox5 expression and TJ degradation was demonstrated. While no differences in blood pressure or locomotor activity were observed, chronic Nox5 expression induced Cox2 upregulation in the brain and induced cognitive deficits in knock-in aged mice [54].

In the following sections, we will first present the cellular constituents of the BBB and discuss our current understanding of the importance of Noxes in cerebral vasculature homeostasis, especially their roles in controlling cerebral vascular tone. We will then describe the main events leading to BBB disruption and the consequent increase in vascular permeability in ischemic stroke. Finally, we will explore how each oxidase has been shown to contribute to BBB dysfunction following ischemic stroke.

### 2.6. The Blood–Brain Barrier and Nox Proteins in Cerebral Vasculature Homeostasis

The BBB is formed by capillary brain endothelial cells surrounded by basal lamina, pericytes and astrocytic perivascular endfeet. The functional and structural integrity of the BBB is not only critical to maintain homeostasis of the brain microenvironment and allow optimal neuronal signaling but also to protect the CNS from injury and disease. This biological barrier protects the brain from potentially harmful compounds found in the bloodstream such as toxins and bacteria, supplies the brain with essential nutrients and mediates efflux of many waste products. The BBB also forms an interface between the peripheral and central immune systems, regulating leukocyte access to brain and contributing to regulation of the innate immune response [55].

Brain endothelial cells that form the walls of the capillaries are polarized, having an apical domain and a basolateral domain, and are tightly sealed by interendothelial junctional complexes. These cells are central to the barrier properties of the BBB. Very specific features distinguish the brain endothelium from the endothelium of peripheral tissues. Transendothelial electrical resistance (TEER), which is typically 2–20 hm·cm^2^ in peripheral capillaries, might be as high as >1000 hm·cm^2^ in the brain endothelium [56], emphasizing the importance of the brain endothelial cells in forming and sealing the BBB.

Junctional complexes between adjacent brain endothelial cells are essential to establish and maintain endothelial barrier function by significantly restricting the paracellular diffusion through the intercellular cleft. The tight junction (TJ) complex is formed by transmembrane proteins including occludin, claudins and junctional adhesion molecules JAM-A, JAM-B and JAM-C. These transmembrane proteins interact with scaffolding proteins of the junctional complex such as zonula occludens protein 1 (ZO-1), ZO-2 and ZO-3 and the Ca^2+^-dependent serine protein kinase (CASK), MAGI-1, MAGI-2 and MAGI-3 (membrane-associated guanylate kinase with inverted orientation of protein–protein interaction domains); the partitioning defective proteins PAR3 and PAR6; and MUPP1 (multi-PDZ protein1) that anchor the complex to the actin cytoskeleton [57,58].

Pericytes are also closely associated with the brain endothelium and play supporting roles in endothelial cell barrier maintenance and function [59]. Pericytes cover approximately 30% of the vascular surface of brain capillaries [60,61] and have an important role in supporting the structural integrity of the BBB [60]. In vivo pericyte loss results in downregulation of ZO-1 and occludin protein levels, vascular permeability and a progressive age-dependent vascular-mediated neurodegeneration [62]. More recently, transcriptomic profiles of human endothelial cells derived from hematopoietic stem cells co-cultured with human brain pericytes revealed that pericytes induce endothelial cell barrier tightening and upregulation of several typical BBB genes such as claudin-3, claudin-7 and claudin-10 [63]. Importantly, because of their contractile nature, pericytes are able to respond to brain-generated vasoactive signals and therefore are critically involved in cerebral blood flow regulation [64,65].

ROS are critically involved in physiologic vasodilator responses within the cerebral vasculature [66,67]. Early studies showed that arachidonate and bradykinin-induced cerebral arteriolar dilation were prevented in in the presence of SOD and catalase [68]. Similarly, a different study performed in rabbits and cats found that dilation of pial arterial vasculature induced by arachidonic acid was prevented by SOD and catalase [69]. However, in many other conditions, ROS, and especially superoxide, impair vasodilation, similar to the situation in systemic arteries [70]. The concentration and composition of ROS seems to determine whether they cause vasoconstriction or vasodilation [71]. While studies on the role of Noxes in BBB homeostasis and brain vasculature are very limited, Nox proteins are an important source of ROS within the cerebral vasculature. When compared to systemic arteries, striking differences with regard to baseline expression of Nox proteins in the cerebral vasculature have been reported. Nox1 and Nox4 mRNA levels are significantly higher than Nox2 in the brain endothelium [72]. Superoxide production (baseline or stimulated by NADPH or angiotensin II) in basilar or middle cerebral arteries (MCA) was 10- to 100-fold greater than in aorta, carotid, renal or mesenteric arteries. Nox activity along with Nox4 expression levels are higher in the basilar artery when compared to these other extracranial arteries. Importantly, activation of Nox in cerebral arteries leads to relaxation and inhibits angiotensin II-induced constriction [66,67]. In contrast, superoxide derived from Nox2 contributes to cerebrovascular dysfunction in multiple diseases such as Alzheimer’s Disease, hypertension, and aging [70]. Thus, it appears that Nox-derived ROS play an important physiologic role in the control of cerebral vascular tone. While it is not clear which BBB cell types are involved, ROS production was detected in brain endothelial cells and vascular smooth muscle cells in basilar arteries [66].

The specialized foot-processes of perivascular astrocytes form a complex network surrounding the brain capillaries and this close association is also critical for the formation and maintenance of the BBB properties. During hemostasis, astrocytes secrete a wide range of angiogenic factors, including transforming growth factor-β (TGFβ), glial-derived neurotrophic factor (GDNF), basic fibroblast growth factor (bFGF) and angiopoetin 1 that induce endothelial cell proliferation, help to maintain an optimal BBB phenotype, regulate cerebral blood flow and enhance the barrier properties of endothelial cells [73]. Astrocyte- and pericyte- mediated GDNF release increased both TEER and the expression of claudin-5 in cultured human brain microvascular endothelial cells [74]. Several studies have also implicated the SHH signaling pathway as an important mechanism employed by perivascular astrocytes to maintain BBB integrity. Astrocytes also perform a protective role by regulating leukocyte transit across the BBB. Studies in vivo and in cultured human astrocytes demonstrated that astrocytes exposed to inflammatory stimuli produce structural TJ complexes, upregulate claudin-1, claudin 4 and JAM-A, and use their own TJ proteins to confine activated T lymphocytes in distinct clusters, restricting leukocyte entry into the perivascular space [75]. Through a mechanism that involves upregulation of protease inhibitors, recently published observations also highlight the importance of astrocytes in protection against degradative enzymes secreted by the infiltrating lymphocytes [76]. Collectively, these findings provide evidence that astrocytic glia limitans represent a coordinated barrier to regulate brain endothelial cell tightness and leukocyte infiltration into the CNS.

### 2.7. Blood–Brain Barrier Disruption in Ischemic Stroke

Stroke is the second leading cause of death and the third-leading cause of death and disability worldwide [77]. Ischemic stroke remains the most common type of stroke and is caused by either a thrombotic or embolic event resulting in decreased cerebral blood flow [78]. While intravenous thrombolysis and mechanical thrombectomy lead to a substantial improvement in outcomes in selected patients, stroke management still represents a significant burden on the healthcare system. During cerebral ischemia, BBB disruption and the consequent increase in vascular permeability nearly always leads to brain edema formation. Brain edema is a life-threatening complication of ischemic stroke, and it is associated with increased intracranial pressure, midline structure deviation, longer hospitalization and has a critical impact on patient morbidity and mortality [79,80].

When cerebral blood flow is interrupted, the delivery of oxygen and glucose—which are essential for CNS metabolism—is compromised. As a consequence, adenosine triphosphate (ATP) levels are reduced and the function of ionic transporters Na^+^-K^+^-ATPase and Ca^2+^-ATPase is impaired, leading to Na^+^ accumulation and cytotoxic edema formation [81]. This process is characterized by cellular swelling and takes place in all CNS cell types, but it is initiated and particularly prominent in astrocytes following ischemia [82].

A progressive endothelial dysfunction that is initiated by ionic edema formation is triggered by transcapillary influx of ions, including and Na^+^, Cl-, and water. The transport of Na^+^ through the luminal membrane and then through the abluminal membrane of brain endothelial cells generates an electrical gradient for Cl- and an osmotic gradient for water. This two-step transport process replenishes and supplies the brain interstitium with additional Na^+^. At this stage, early water influx only correlates with Na^+^ accumulation [83]. Endothelial transporters and channels that mediate ionic edema formation involve Cl^−^ co-transporters such as NKCC1 and KCC, sodium-glucose linked transporter 1 (SGLT1) and glucose transporter 1 (GLUT1) among others [82]. Importantly, capillary structural integrity is maintained during ionic edema formation [83].

**Figure 2:**
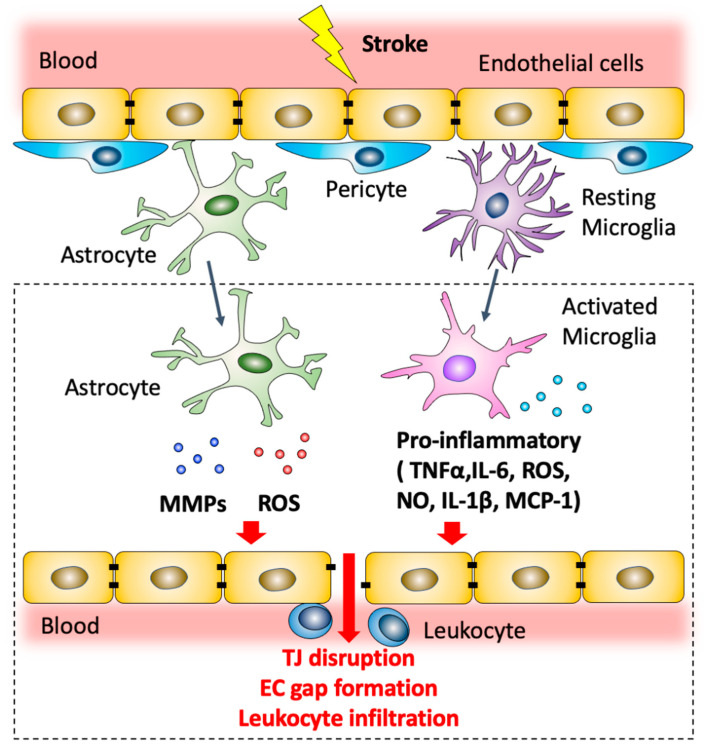
Modulation of blood–brain barrier permeability by astrocytes and microglia after ischemic stroke. Post-ischemic astrocytes produce MMPs and ROS that contribute to TJ disruption and endothelial cell gap formation. Microglial cells are polarized to a pro-inflammatory phenotype. Pro-inflammatory microglia can produce a variety of mediators including NO, ROS and pro-inflammatory cytokines, including TNF-α, IL-1β and IL-6, which contribute to downregulation of junctional complexes between adjacent brain endothelial cells and lead to BBB dysfunction. Activated microglial cells also secrete MCP-1 which promotes leukocyte recruitment.

Within minutes after cerebral ischemia onset, microglial cells are activated, and a robust inflammatory response is then initiated. Activated microglial cells can produce a variety of mediators including nitric oxide (NO), ROS and pro-inflammatory cytokines, including TNF-α, IL-1β and IL-6, which contribute to downregulation of junctional complexes between adjacent brain endothelial cells and lead to BBB dysfunction (Figure 2). Chemoattractants such as monocyte chemoattractant protein-1 (MCP-1) that promote directed migration of leukocytes are also released, initiating leukocyte recruitment [84]. Activation of perivascular astrocytes has been reported to induce matrix metalloprotease-9 (MMP-9) expression, ROS formation, and cytokine production, leading to redistribution and downregulation of TJ proteins in endothelial cells and to an initial BBB disruption phase [85,86].

Inflammation driven by activation of microglia and astrocytes then triggers a second phase of endothelial dysfunction. Inflammation mediated-actin-dependent endothelial cell contraction and retraction leads to paracellular permeability and subsequent increase in BBB permeability (Figure 2). This event contributes to the initiation of vasogenic edema formation. Vasogenic edema is characterized by extravasation of fluids that contain plasma proteins and is a critical step in the formation of brain edema and swelling [82].

### 2.8. NADPH Oxidases and BBB Dysfunction in Ischemic Stroke

ROS have been shown to induce brain endothelial barrier dysfunction by their ability to lead to downregulation and functional modifications of junctional complexes [87,88]. Nox family members and the ROS they produce have been identified as critical contributors to endothelial dysfunction and BBB permeability induced by cerebral ischemia and several other CNS pathologies such as experimental autoimmune encephalomyelitis and traumatic brain injury [89]. Nox2 is the most extensively studied of the Nox members in the context of BBB dysfunction in ischemic stroke. Studies using Nox2 knockout mice have suggested that these mice are protected against BBB permeability increases induced by transient MCAO (tMCAO), while lesion volume was also significantly attenuated [90]. In a different study, Nox2 knockdown prevented increases in BBB permeability and MMP-9 activity as well as occludin downregulation after tMCAO. This suggests that Nox2 might have a role in MMP-9 upregulation, mediating the proteolytic degradation to the BBB in ischemic stroke [91]. Moreover, treatment with apocynin, an inhibitor of p47phox-containing NADPH oxidases and an antioxidant, reduced MMP-9 activation, consistent with a link between Nox2-derived ROS and MMP-9 induction [92]. Brain endothelial cells express a functionally active Nox2, and hypoxia and reoxygenation induce Rac-1 translocation to the membrane, which is a step required for Nox2 activation and initiation of superoxide production [90]. Remarkably, in an in vitro model of BBB comprising human brain endothelial cells and astrocytes subjected to oxygen and glucose deprivation to mimic in vivo ischemia, apocynin treatment attenuated endothelial permeability and superoxide production through a mechanism that involves modulation of MMP-2 and tissue-type plasminogen activator (tPA) [93].

The contribution of isoform Nox1 to BBB permeability induced by cerebral ischemia has also been demonstrated. There are, however, relatively few studies of the role of Nox1 in brain endothelial permeability and BBB dysfunction and the role of Nox1 in cerebral ischemia is still controversial. It was shown that in vivo Nox1 depletion reduces neurological deficits, lesion volume and BBB disruption in a cerebral ischemia model induced by tMCAO [90]. In a different study, in the absence of Nox1, no protection was observed either in terms of infarct volumes or on functional outcomes following tMCAO [94]. Studies suggest that high levels of Nox1 are expressed in brain endothelial cells [72] but very little is known about the signaling pathways mediated by Nox1 in these cells. In vitro studies using brain endothelial cells subjected to oxygen and glucose deprivation have shown that treatment with a Nox1 inhibitor resulted in reduced ROS production, increased occludin expression and, consequently, reduced endothelial monolayer permeability [95].

A number of different studies have identified a role of Nox4 in BBB dysfunction. Nox4 mRNA levels were found to be upregulated as early as 24 h after cerebral ischemia induction in mice and persisted throughout 30 days, peaking at days 7 and 15 [2]. In both tMCAO and permanent MCAO models, Nox4 knockdown prevented oxidative stress, neuronal apoptosis and BBB permeability [94]. Nox4 knockout mice subjected to tMCAO developed significantly smaller brain infarct sizes when compared with wild-type mice. Furthermore, endothelial-specific Nox4 deletion attenuated BBB permeability as evaluated by Evans blue dye extravasation 24 h after tMCAO induction [40]. More recently, in an intracerebral hemorrhage stroke model, siRNA-mediated Nox4 knockdown improved neurobehavioral scores and prevented neuronal apoptosis and BBB dysfunction [96]. Mechanistically, microRNA-92b regulation of Nox4 expression contributes to viability and permeability induced by oxygen and glucose deprivation in cultured brain endothelial cells [97]. Small interfering RNA-mediated Nox4 knockdown or treatment with GKT137831, a putative Nox1/Nox4 inhibitor, inhibited TNF-α- and glutamate-induced ROS formation and apoptosis in primary cultures of cerebral microvascular endothelial cells [98,99]. However, GKT137831 has recently been reported to interfere with peroxidase-dependent assays of ROS, leading to questions concerning its specificity. In a different study, Nox4 depletion prevented oxygen and glucose deprivation-induced apoptosis and ROS generation in cultured rat brain microvascular endothelial cells. Moreover, the long noncoding RNA MEG3 has been shown to inhibit apoptosis through p53-mediated Nox4 induction [100]. Taken together, these studies suggest a critical role for Nox4 in maintenance of the BBB in the setting of cerebral ischemia.

The involvement of Nox5 in BBB dysfunction induced by cerebral ischemia has also been described recently. Using humanized Nox5 knock-in mice, it was demonstrated that Nox5 leads to increased stroke volume and BBB permeability, and aggravates motor function 24 h after tMCAO induction. The same study confirmed substantial Nox5 expression in brain endothelial cells, as well as increased ROS production 24 h after stroke induction in Nox5 knock-in mice, when compared to control wild-type animals [53]. Indeed, specific Nox5 expression in endothelial cells leads to BBB permeability and cognitive deficits in aged mice, which supports the role of endothelial Nox5 in BBB dysfunction [54].

## 3. Conclusions

On the basis of existing literature, it is clear that NADPH oxidases are essential to normal cerebral vascular function and participate in the progression of BBB damage induced by cerebral ischemia. In physiological conditions, Nox-derived ROS mediate signaling pathways involved in cerebral vascular tone. In cerebral ischemia, these enzymes contribute to increased BBB permeability by mediating MMPs activation, downregulation of TJ proteins and brain endothelial cell apoptosis. Consequences of increased BBB permeability following ischemic stroke include movement of water, plasma proteins and leukocyte extravasation into the CNS, leading to additional tissue damage, and edema formation. Brain edema is a is a major cause of increased morbidity and disability are among survivors. Therefore, there is a critical need for therapeutic strategies to target BBB dysfunction and brain endothelial cells permeability. Therapeutic interventions that target NADPH oxidases may have beneficial effects in cerebral vascular diseases associated with BBB dysfunction such as ischemic stroke. However, additional studies are needed in order to better understand how each one of the NADPH oxidases impacts signaling pathways involved in endothelial barrier function including TJ degradation and cytoskeleton remodeling.

## Data Availability

Not applicable.
